# Disparities in direct access to acute geriatric care – an observational analysis of geographic variations of ambulance conveyance

**DOI:** 10.1186/s12877-026-08370-1

**Published:** 2026-10-02

**Authors:** Sofi Varg, Veronica Vicente, Matilda Hagman, Naimi Johansson, Peter Lindgren, Emelie Heintz, Clas Rehnberg

**Affiliations:** 1 Department of Learning, Informatics, Management and Ethics, Centre for Health Economics & Policy, Karolinska Institutet, Stockholm, Sweden; 2 Centre for Health Economics, Informatics and Health Services Research, Stockholm Health Care Services, Stockholm, Sweden; 3 Ambulance Medical Service in Stockholm [Ambulanssjukvården i Storstockholm AB], Stockholm, Sweden; 4 Department of Clinical Science and Education, Karolinska Institutet, Stockholm, Sweden; 5 The Swedish Institute for Health Economics, Lund, Sweden

**Keywords:** Access to healthcare, Geographic variation, Older adults, Geriatric care, Emergency medical services, Prehospital care

## Abstract

**Background:**

Ambulance services often represent a key entry point to healthcare for older adults, and subsequent care pathways have important implications for outcomes and resource use. Direct admission to geriatric units may offer benefits for certain patients, but access varies. This study aims to examine whether geographic area of residence is associated with ambulance conveyance to geriatric units as an alternative to emergency departments among older adults and to explore patient- and system-level factors associated with observed variation.

**Methods:**

A cross-sectional, register-based study of ambulance assignments of adults ≥ 65 years in Stockholm, Sweden (2017–2019) was conducted. Eight geriatric units, each representing a distinct hospital service area, were included. The outcome was direct admission to a geriatric unit versus to an emergency department. Geographic variation was assessed using random intercept multilevel logistic models, adjusting for demand-side and supply-side factors, with measures of variation including intraclass correlation coefficient (ICC), median odds ratio (MOR), and proportional change in variance (PCV).

**Results:**

Among 8208 patients included in the study, 603 (7%) were directly admitted to a geriatric unit. Crude conveyance rates across hospital service areas ranged from 4% to 21%. The unadjusted between-area variance (τ²) was 0.3510 (ICC = 0.096). When supply-side factors were assessed individually while controlling for demand, proximity to a geriatric unit, geriatric unit ownership, and number of staffed beds resulted in estimated reductions in the between-area variance by 26%, 27%, and 38%, respectively, suggesting that these factors may contribute to the observed area-level heterogeneity. In the fully adjusted model, τ² decreased by 41% to 0.2076 (ICC = 0.059), although considerable residual heterogeneity remained (MOR = 1.54), indicating that otherwise similar patients may face different probabilities of direct geriatric admission depending on their hospital service area. Robustness analysis with a Bayesian approach revealed large uncertainty around the estimated variance.

**Conclusions:**

Direct ambulance conveyance to geriatric units varies substantially across hospital service areas. While proximity to units and greater geriatric bed capacity likely had an association with reduced between-area variation, meaningful residual differences persist, suggesting that unmeasured factors beyond patient need and measured supply characteristics may influence access to acute geriatric care.

**Supplementary information:**

The online version contains supplementary material available at https://doi.org/10.1186/s12877-026-08370-1.

## Background

For older adults requiring unscheduled medical care, ambulance services often constitute an important entry point into the healthcare system, as older individuals represent a substantial proportion of emergency medical service (EMS) users [1–3]. The care pathway following ambulance assessment, most commonly conveyance to an emergency department (ED), has important implications for healthcare utilisation, patient outcomes, and system efficiency [4–6].

Older patients frequently present with complex health states characterised by multimorbidity, frailty, and geriatric syndromes such as falls, delirium, and functional impairment [7]. These conditions are associated with a need for comprehensive and multidisciplinary assessment rather than single-condition–focused management [8], [9]. EDs are primarily designed for rapid assessment and treatment of acute conditions. Previous studies have shown that older adults in ED settings are at increased risk of long waiting times, fragmented care, and functional decline, particularly when presenting with complex care needs [10–12]. Consequently, ED care may be suboptimal for selected older patients whose primary needs are not strictly emergency-driven.

Geriatric units (GUs) offer specialised care models incorporating comprehensive geriatric assessment and interdisciplinary management. Such models can be associated with improved outcomes, including reduced institutionalisation, and improved functional status, compared with usual care pathways [13–15]. Studies from France have further suggested that direct admission to GUs, bypassing the ED, may reduce length of stay and post-acute transfers and potentially be cost-effective for selected older patients [5], [6]. In Sweden, a prehospital decision system in the EMS for older adults has shown that patients receiving direct care at a GU rather than via the ED express satisfaction with their care [16], and typically remain at the GU for the duration of their care episode, thereby bypassing the ED and avoiding intra-hospital transfers [17].

Despite these findings, access to direct geriatric admission following ambulance care remains inconsistent. To identify effective practices and areas for improvement, it is essential to examine the determinants influencing decision-making across GUs when admitting patients to the unit. This could be differences in both patient characteristics, often addressed as demand-side factors, and in healthcare delivery, referred to as supply-side factors, which may vary due to e.g. variations in staffing, technical infrastructure, and service availability [18], [19]. In previous studies addressing geographical variation in healthcare spending, it has proven difficult to empirically sort out the determinants of variation and there is conflicting evidence with regards to explaining factors [20–25]. Rather, the determinants of variation appear to depend on the specific healthcare setting, the outcome measure, and the level of geographical aggregation, which underscores the importance of studying each context to effectively target healthcare improvements. However, all geographic variation is not inherently problematic. Differences in healthcare utilization may reflect legitimate variations in population characteristics or demand factors such as age, sex, and health status [18]. In the context of ambulance conveyance to EDs in the US, the greatest variability across hospital referral regions has been attributed to differences in health status, followed by socioeconomic factors, with provider availability contributing to a lesser extent [26]. While some variation is justified by medical need, reducing unwarranted geographic disparities is essential for improving healthcare system efficiency [18].

This study aims to examine whether geographic area of residence is associated with ambulance conveyance to geriatric units as an alternative to emergency departments among older adults and to explore patient- and system-level factors associated with observed variation.

## Methods

### Study design

This register-based cross-sectional study covered the period from January 1, 2017 to December 31, 2019. Ethical approval was obtained from the Regional Ethics Review Board in Stockholm (reference numbers 2016/2373-31/5 and 2022-03943-02).

### Study setting

The study was conducted in Stockholm, Sweden’s most populous region, with approximately 2.3 million inhabitants in 2017–2018 [27] . Sweden has a universal, tax-funded healthcare system with shared responsibility across the state, regions, and municipalities [28]. The state defines the overall framework, while the 21 regions organize and finance somatic and psychiatric care at primary, secondary, and tertiary levels. Municipalities (*n* = 290) are responsible for social services and long-term care for older adults and individuals with functional impairments. Healthcare services, including ambulance care, are largely publicly funded, with patients paying nominal subsidized fees. Specialized somatic care accounts for the largest share of healthcare expenditure (47%). Although care is delivered by both public and private providers, all accredited providers operate under the same regulatory and reimbursement systems [28].

GUs provide secondary care through interdisciplinary teams, primarily targeting older adults with complex health needs who require rehabilitation or continued medical care following acute hospitalization. These units are located either within acute hospitals or at smaller hospitals. During the study period, 12 GUs in Stockholm accepted direct admission from ambulances; however, units integrated within acute hospitals (*n* = 4) were excluded due to data limitations. The study therefore includes eight GUs (two public, six private), each serving a distinct hospital service area (HSA). In this study, the HSAs serve as geographic units, with patients assigned based on their area of residence. Each HSA included 6000–50 000 residents aged ≥ 65 years. Patients were mutually exclusive to one HSA.

The ambulance services within the EMS provide prehospital care and are staffed by registered and specialized nurses. Organizational support and guidelines assist in determining appropriate levels of emergency care [29], [30]. During the study period, ambulance services were provided by one public and two private providers, with geographically unrestricted ambulance dispatching in Stockholm. In Stockholm, a prehospital decision system directing older adults to alternative care destinations has been in use in the EMS since 2011. The decision system includes a decision support tool that guide older patients with specific conditions and mild acute illnesses toward alternative care pathways, including direct conveyance to GUs [31], [32]. The decision to convey a patient to the GU is taken in concordance with the patient and the doctor on call at the GU [31], [23].

### Study population

**Fig. 1:**
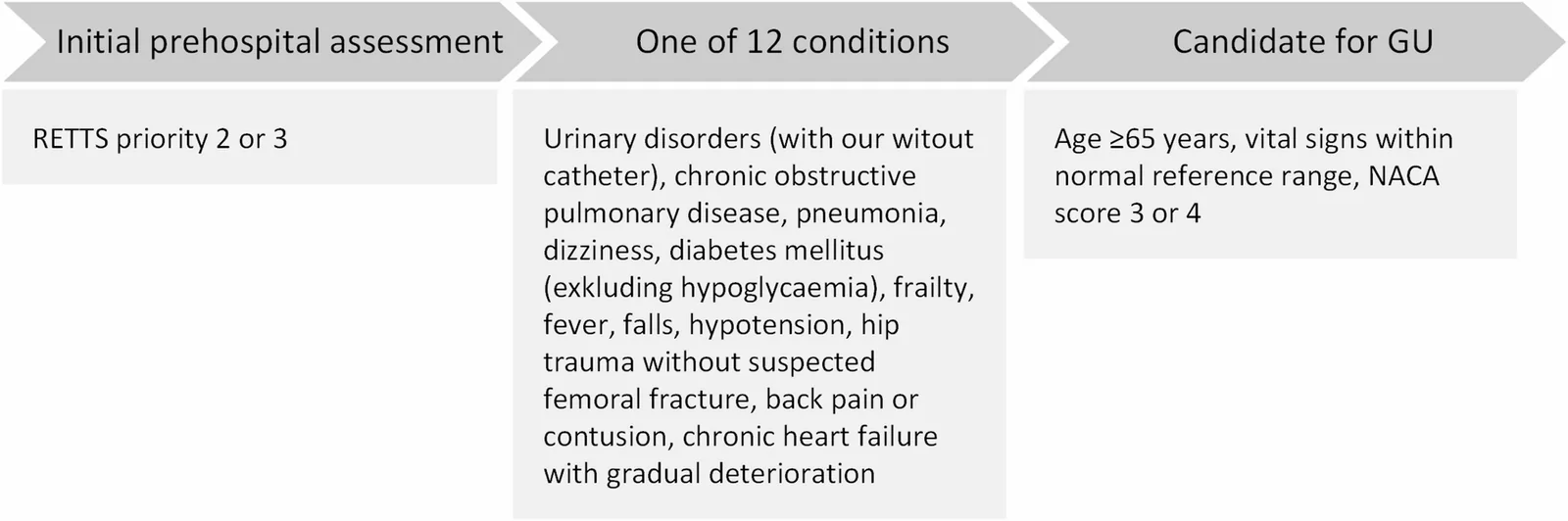
Eligibility criteria for direct admission at a geriatric unit

The study population included all patients transported by ambulance during the study period who met criteria for direct prehospital conveyance to GUs. Eligible patients were aged ≥ 65 years, classified as having urgent but non-life-threatening conditions (RETTS priority level 2 or 3), had vital signs within normal reference ranges, and a National Advisory Committee for Aeronautics (NACA) score indicating a condition requiring inpatient care or potentially life-threatening illness (NACA 3 or 4) [32]. In addition, patients had to present with one of twelve predefined conditions considered suitable for prehospital geriatric assessment and alternative care pathways [32] (Fig. 1).

Due to limitations in register data, exact clinical conditions could not be identified. Instead, Emergency Symptoms and Signs (ESS) codes, in combination with assessed risk levels and vital signs, were used as proxies for clinical condition and severity. This approach may have resulted in some over-inclusion, potentially leading to an underestimation of the crude conveyance rate to GUs.

The analysis was restricted to patients residing in the Stockholm Region and transported within the region by ground ambulance to either GUs or EDs. Inter-hospital transfers were excluded. Transports occurring outside the patient’s assigned HSA were excluded to ensure mutually exclusive geographic clusters, resulting in exclusion of 1.13% of the sample. For patients with multiple ambulance assignments during the study period, only the first assignment was included as the index event, leading to exclusion of subsequent events for approximately 10% of individuals.

### Study outcome

The primary outcome was direct admission to a GU, defined as a binary variable (1 = admission to GU; 0 = non-admission, i.e., admission to the ED). Admission status was determined based on the recorded ambulance destination.

### Data sources and variables

Individual-level data were obtained from the Stockholm Regional Healthcare Database, which contains pseudonymized records of all regionally financed healthcare services provided by both public and private providers. Area-level data were sourced from the Health and Medical Care Administration in Stockholm, Sweden. Several individual-level variables on the demand-side were included in the analysis to account for variation in healthcare needs across areas. These variables were selected to capture characteristics that may influence both healthcare needs and eligibility for direct admission to a GU, including age, sex, lifestyle/socioeconomic status (SES), living conditions, comorbidity burden and previous healthcare utilisation. To capture variation in healthcare supply, several structural and organizational factors were considered. Factors on the supply-side were included both at individual level and at HSA level, and were chosen to reflect differences in accessibility, availability, and organization of geriatric care that may influence conveyance decisions and access to direct admission to GU pathways. Included variables are presented in Table 1.

**Table 1:** Study variables included in the analysis

Domain	Variable	Level	Definition/Categorization
**Demand side** **(patient-level)**	**Age**	Individual	65–74; 75–84; 85–94; ≥95 years
	**Sex**	Individual	Male / Female
	**Lifestyle/ Socioeconomic status (SES)**	Small area level (individual assigned)	Composite index (Mosaic) based on area-level income, education, living arrangements, and sociodemographic characteristics; categorised as highly advantaged; medium advantaged; disadvantaged
	**Living situation**	Individual	Living at home / Living in institution
	**Comorbidity**	Individual	Charlson Comorbidity Index (CCI) categorised as 0 (no comorbidity); 1–2 (mild comorbidity); 3–4 (moderate comorbidity); ≥5 (severe comorbidity)
	**Days hospitalized previous year**	Individual	Total number of inpatient days in previous 12 months. Categorised as 0 days (none); 1–13 days (Less than two weeks); ≥14 days (Two weeks or more)
**Supply-side** **(healthcare system)**	**Admission time**	Individual	Admission to geriatric unit during office hours (Monday-Friday 08–17) vs. outside office hours
	**Geographic accessibility**	Individual	Straight-line distance (km) from patient area of residence to assigned GU relative to distance to assigned ED. Categorised as GU closer; ED closer; equal distance (both facilities within 5 km).
	**GU ownership**	HSA	Public / Private
	**Geriatric bed capacity**	HSA	Number of staffed geriatric unit beds per 1000 residents aged ≥ 65 years

### Statistical analysis

Descriptive statistics are presented as either proportion in percent, or mean value, by admission unit (GU or ED). The range of corresponding values over HSAs are moreover presented with minimum to maximum value.

Data have a multilevel structure with patients nested in HSAs. As the outcome is binary, a multilevel logistic regression using residual subject-specific pseudo-likelihood (RSPL), a penalized quasi-likelihood based estimation, was applied to address differences between HSAs with regards to direct admittance to GUs. The models included random intercepts for HSAs, allowing the probability of direct admission to a GU to vary across HSAs.

Estimation of multilevel logistic regression models may be challenging when the number of clusters is small because variance components and their standard errors can be biased and imprecisely estimated [33]. No estimation method has been shown to perform uniformly well under such conditions, and the relative performance of alternative estimators depends on both the number and size of clusters. Given the small number of HSA clusters (*n* = 8) and the relatively large cluster sizes, RSPL was selected as the primary estimation approach based on the argument that the estimation method may perform favourably when the number of clusters is small but cluster sizes are relatively large [33].

Seven separate models (M) were built. First, a baseline model (M0) was constructed that only included a random intercept. M1 additionally included individual characteristics (*age*,* sex*,* lifestyle/SES*,* living situation*,* days hospitalized previous year*, and *comorbidity*). M2-M3 incorporated M1 and additionally included supply-side determinants measured at an individual level separately (*admission time* and *geographic accessibility*). M4-M5 added supply-side determinants measured at an HSA level separately (*GU ownership* and *geriatric bed capacity*). The last model (M6) incorporates all individual characteristics and supply-side determinants. The statistical equations for the models are shown below:

0. $$logit\left(P\right({Y}_{ij}=1\left)\right)={\beta\:}_{0}+{u}_{j}$$
1. $$\:logit\left(P\left({Y}_{ij}=1\right)\right)={\beta\:}_{0}+{X}_{ij}\beta\:+{u}_{j}$$
2. $$\:logit\left(P\left({Y}_{ij}=1\right)\right)={\beta\:}_{0}+{X}_{ij}\beta\:+{\gamma\:}_{1}{Z}_{ij}^{\left(1\right)}+{u}_{j}$$
3. $$\:logit\left(P\left({Y}_{ij}=1\right)\right)={\beta\:}_{0}+{X}_{ij}\beta\:+{\gamma\:}_{2}{Z}_{ij}^{\left(2\right)}+{u}_{j}$$
4. $$\:logit\left(P\left({Y}_{ij}=1\right)\right)={\beta\:}_{0}+{X}_{ij}\beta\:+{\delta\:}_{1}{W}_{j}^{\left(1\right)}+{u}_{j}$$
5. $$\:logit\left(P\left({Y}_{ij}=1\right)\right)={\beta\:}_{0}+{X}_{ij}\beta\:+{\delta\:}_{2}{W}_{j}^{\left(2\right)}+{u}_{j}$$
6. $$\begin{array}{lll}logit\left(P\left({Y}_{ij}=1\right)\right)={\beta}_{0}+{X}_{ij}\beta+{\sum}_{k=1}^{2}{\gamma}_{k}{Z}_{ij}^{\left(k\right)}\\ \qquad\qquad\qquad\qquad\quad+{\sum}_{k=1}^{2}{\delta}_{k}{W}_{j}^{\left(k\right)}+{u}_{j}\end{array}$$      

where *Y*_*ij*_ is the binary outcome for individual *i* in HSA *j* and *logit(P(Y*_*ij*_*=1))* is the log-odds of the outcome. *β*_*0*_ is the overall intercept and *u*_*j*_ is the random intercept for HSA *j*. *X*_*ij*_*β* is the combined effect of individual-level demand-side factors where *X*_*ij*_ is the vector of these variables and *β* the corresponding vector of the coefficients. $\:\sum\:_{k=1}^{2}{\gamma\:}_{k}{Z}_{ij}^{\left(k\right)}$ represents the supply-side factors measured on individual-level where each $\:{Z}_{ij}^{\left(k\right)}$ is a different supply-side variable and $\:{\gamma\:}_{k}$ is its coefficient. The supply-side factors measured on HSA-level are represented by $\:\sum\:_{k=1}^{2}{\delta\:}_{k}{W}_{j}^{\left(k\right)}$ where each $\:{W}_{j}^{\left(k\right)}$ is a different HSA-level variable and $\:{\delta\:}_{k}$ is its coefficient.

Measures of variation were computed to assess HSA variation. The intraclass correlation coefficient (ICC) was used to measure to what extent the total variation in the probability of direct admission to GU that was accounted for by living in certain HSAs. As a logistic multilevel model was used, the logistic approximation with π^2^/3 ≈ 3.29 was applied, where τ^2^ represents the random effects estimated variance distribution [34], [35].

$$\:ICC=\frac{{\tau\:}^{2}}{{\tau\:}^{2}+\frac{{{\uppi\:}}^{2}}{3}}$$

Furthermore, to quantify the variation between clusters and translate the area level variance into an odds ratio scale, the median odds ratio (MOR) is presented as a measure of heterogeneity. The MOR quantifies the median increase in odds of direct admission to GU when comparing two identical individuals from two randomly selected clusters, reflecting the extent of between-cluster heterogeneity [34], [36]. In the formula, the Φ^−1^(0.75) = 0.6745 is the 75th percentile of a standard normal distribution [34].

$$\:MOR=exp\left(\sqrt{2{\tau\:}^{2}}\times\:{\varPhi\:}^{-1}\left(0.75\right)\right)$$

To address how the variation differs between the models, the proportional change of variance (PCV) is presented. The PCV represents the proportion of between-cluster variance explained by the covariates introduced in subsequent models, calculated by comparing the cluster-level variance of an adjusted model to that of the baseline model (M0). To isolate the effect of the supply-side variables, PCV is additionally calculated for subsequent models (M2-M4) relative to the model adjusting for demand-side characteristics (M1).

Data was analysed with SAS^®^ Enterprise Guide^®^ software, version 8.6 [37] and R software [38] was used to quantify the measures of variation.

### Robustness analyses

To evaluate the robustness of the estimated between-HSA variation, three complementary approaches were applied. First, area-specific log-odds were estimated using a fixed-effects logistic regression model without an intercept, and the sample variation of these estimates was calculated. Second, the variance estimate obtained from the unadjusted RSPL-based multilevel model was compared with that obtained using adaptive Gaussian quadrature (AGQ). Third, a Bayesian multilevel logistic regression model with default weakly informative priors was fitted. Variance estimates obtained from these alternative approaches were compared to assess the consistency of the estimated between-HSA variation and the sensitivity of conclusions regarding residual geographical variation to the choice of estimation method.

## Results

The study sample consists of 8208 patients, of which 603 patients (approximately 7%) were directly admitted to a GU. The number of patients per HSA ranged from 144 to 1819, and the number of direct GU admissions per HSA ranged from 30 to 178. The crude conveyance rates of direct admission to GUs across included HSAs varied substantially, ranging from 4% to 21%. Descriptive statistics for both demand- and supply-side characteristics are presented in Table 2.

**Table 2:** Study population and area level characteristics by admission unit, and range by clusters proportion

	**Variable**	**Emergency department** (*n* = 7605)	**Geriatric unit** (*n* = 603)	**Cluster range** proportion per HSA(*n* = 8)
**DEMAND**	**Individual level**			
	Age groups			
	65–74 years	27% (2047)	9% (55)	22%-31%
	75–84 years	37% (2824)	33% (196)	34%-44%
	85–94 years	31% (2384)	47% (282)	26%-37%
	≥ 95 years	5% (350)	12% (70)	2%-7%
	**Sex**			
	Female	56% (4284)	62% (371)	51%-60%
	Male	44% (3321)	38% (232)	40%-49%
	**Lifestyle/SES**			
	Highly advantaged area	33% (2510)	27% (164)	5%-78%
	Medium advantaged area	31% (2382)	36% (215)	16%-50%
	Disadvantaged area	36% (2699)	37% (224)	6%-62%
	**Living situation**			
	Home	92% (6978)	96% (581)	90%-97%
	Institution	8% (627)	4% (22)	3%-10%
	**Days hospitalized previous year**			
	None	42% (3207)	26% (157)	38%-45%
	Less than two weeks	25% (1909)	23% (141)	22%-27%
	Two weeks or more	33% (2489)	51% (305)	29%-40%
	**Comorbidity (CCI)**			
	No comorbidity	12% (875)	7% (40)	10%-13%
	Mild comorbidity	32% (2445)	32% (193)	30%-34%
	Moderate comorbidity	26% (1979)	29% (175)	24%-28%
	Severe comorbidity	30% (2306)	32% (195)	28%-33%
**SUPPLY**	**Admission time**			
	Office hours, mon-fri 08–17	47% (3573)	62% (371)	45%-53%
	Out-of-office hours	53% (4032)	38% (232)	47%-55%
	**Geographic accessibility**			
	GU and ED within equal distance	44% (3339)	43% (261)	0%-100%
	ED closer	10% (754)	4% (23)	4%-42%
	GU closer	46% (3503)	53% (318)	9%-100%
	**Health service area level**			
	**GU Ownership**			
	Private	67% (5120)	80% (480)	0%-100%
	Public	33% (2485)	20% (123)	0%-100%
	**Geriatric bed capacity**			
	Beds per 1000 older population, mean (sd)	2.6 (0.53)	2.8 (0.5)	1.6–3.5

On the demand side, patients conveyed to GUs tended to be older, female, more often lived in medium- or disadvantaged areas, had a higher tendency to live at home, and had more prior hospital days and slightly more comorbid disease (Table 2). These characteristics also had a notable variation across HSAs, specifically with regards to socioeconomic area, where the proportion of patients living in for example disadvantageous areas varied from 6% to 62%.

Multilevel modelling showed that the estimated between area variance (τ^2^) was 0.351, corresponding to an ICC of 0.096, implying that around 10% of the total variation in conveyance outcomes was attributable to differences between HSAs (M0, Table 3). Taking demand-side factors into consideration revealed a slight increase in HSA-level variance with almost 9% (M1, Table 3) compared to the baseline model (M0), suggesting that the included demand-side factors did not explain the observed variation but rather accentuated differences between areas. Parameter estimates for the demand-side variables are presented in Additional file 1 (Table A1). Results were similar when estimated using RSPL and AGQ.

Subsequent inclusion of supply-side factors mainly resulted in estimated reductions in the between-area variance. Relative to the baseline model (M0), the PCV ranged from − 9% to 41%, suggesting that several factors may contribute to a substantial proportion of the observed geographic variation (Table 3). When estimating PCV relative to the demand-adjusted model (M1), thereby isolating the contribution of supply-side factors, corresponding PCV estimates were slightly larger, reflecting the reduction in residual variance associated with supply-side factors conditional on demand-side characteristics.

**Table 3:** Multilevel logistic regression models

	M0	M1	M2	M3	M4	M5	M6
**MEASURES OF ASSOCIATION, OR (CI)**							
Intercept (Estimate)	-2.43	-4.42	-4.70	-4.51	-5.03	-6.30	-6.36
Individual characteristics		*NR*	*NR*	*NR*	*NR*	*NR*	*NR*
**INDIVIDUAL LEVEL**							
**Admission time**							
Office hours (vs. out-of-office hours)			1.77* (1.49–2.11)				1.79* (1.50–2.14)
**Geographic accessibility**							
GU closer (vs. equal distance)				1.44* (1.08–1.93)			1.42* (1.06–1.90)
ED closer (vs. equal distance)				0.50* (0.30–0.81)			0.49* (0.30–0.80)
**HSA LEVEL**							
**GU ownership**							
Private (vs. public)					2.27 (0.95–5.46)		1.37 (0.58–3.25)
**Geriatric bed capacity**							
Beds per 1000 older population (continuous)						2.01* (1.09–3.70)	1.61 (0.84–3.07)
**MEASURES OF VARIATION OR CLUSTERING**							
HSA variance, τ² (SE)	0.351 (0.203)	0.382 (0.220)	0.383 (0.220)	0.284 (0.167)	0.280 (0.178)	0.236 (0.148)	0.208 (0.143)
PCV(M0) ^a^	Ref	-8.9%	-9.0%	19.1%	20.2%	32.7%	40.9%
PCV(M1) ^b^		Ref	-0.1%	25.7%	26.7%	38.2%	45.7%
ICC	0.096	0.104	0.104	0.079	0.078	0.067	0.059
MOR	1.76	1.80	1.80	1.66	1.66	1.59	1.54

*p < 0.05

^a^PCV relative to the baseline model (M0)

^b^PCV relative to the demand-adjusted model (M1)

OR= Odds ratio; CI= Confidence interval; NR = Not reported (data available in Additional file 1, Table A1); τ² = tau-squared; SE= Standard error; PCV= Proportional change of variance; ICC=Intraclass correlation; MOR = Median odds ratio

On the supply side, Table 2 shows that the proportion of patients varied substantially between admission units with respect to admission time and GU ownership. At the same time, there was also a notable variation in the proportions between HSAs for geographic accessibility, GU ownership, and geriatric bed capacity (Table 2). Even if being transported during office hours had a significant association with the outcome, it did not explain the HSA-level differences (M2, Table 3).

The supply-side factors geographic accessibility (M3, Table 3), GU ownership (M4, Table 3) and geriatric bed capacity (M5, Table 3), were associated with estimated reductions in the between-area variance with 26%, 27% and 38%, respectively, relative to the demand-adjusted model (M1), suggesting that these area-level factors may contribute to the observed area-level heterogeneity.

**Fig. 2:**
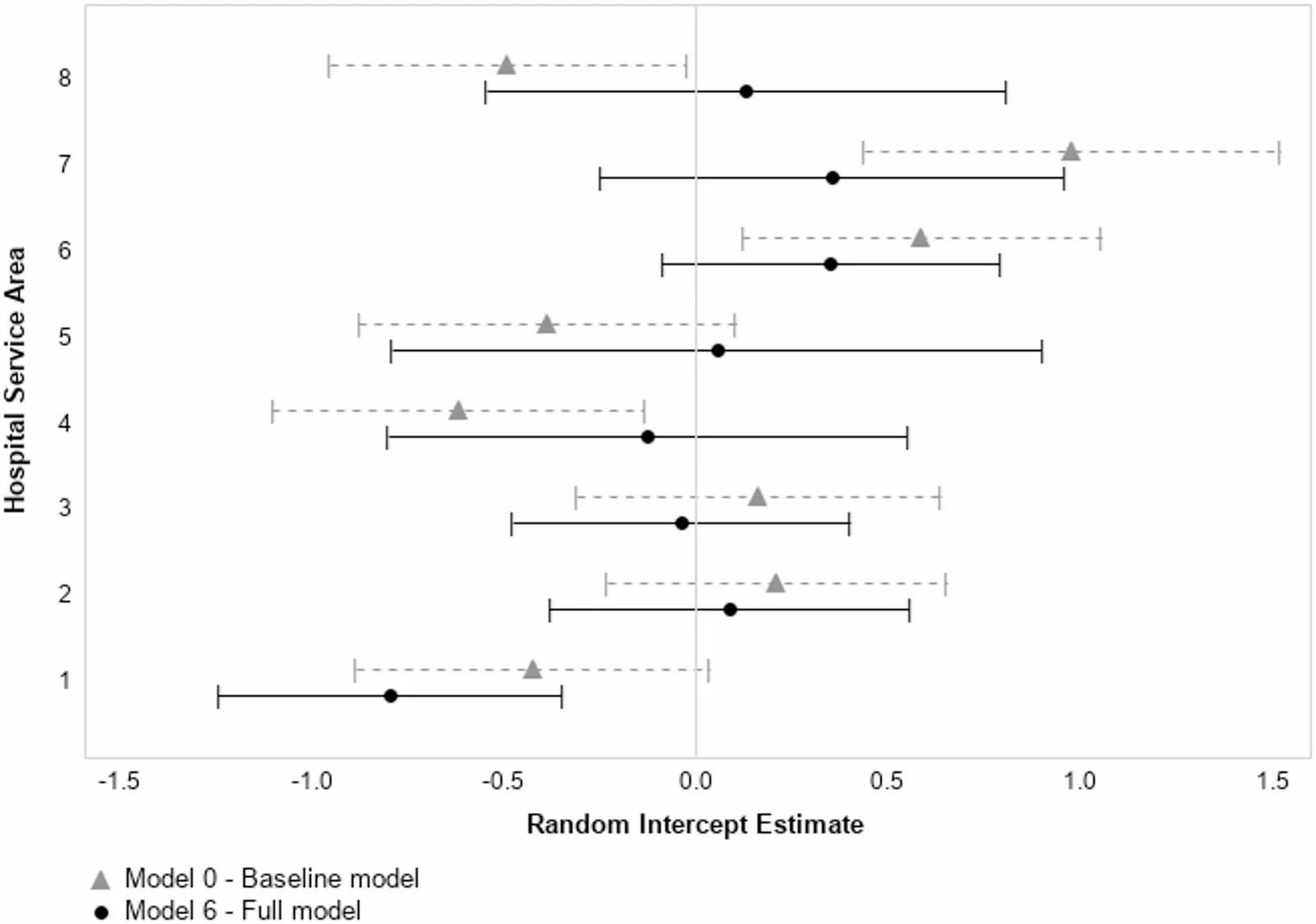
Random intercept estimates (CI 95%) for Model 0 and Model 6 for each HSA respectively

After adjusting for all included demand- and supply-side factors (M6, Table 3), the estimated HSA-level variance was 0.2076, corresponding to a 41% reduction relative to the baseline model (M0), with an ICC of 0.059. Despite this reduction, the MOR of 1.54 indicates that residual geographic variation persists. These notable differences in the likelihood of direct admission to GUs across HSAs are shown in Fig. 2.

The overall pattern of variation is largely consistent with the unadjusted model (Fig. 2, M6 compared to M0), indicating that certain HSAs remain more prone to direct admission to GUs even after accounting for individual- and area-level covariates. Visualisations of M1-M5 are presented in Additional file 1 (Figure A1-A5).

Robustness analyses were performed for the baseline model (M0) using alternative estimation approaches. The variance of the area-specific log odds from the fixed-effects estimate was 0.39, while the estimated between-HSA variance ranged from 0.30 to 0.35 in the frequentist multilevel models, depending on estimation method used. Full model estimates obtained using AGQ are presented in Additional File 1 (Table A2) and showed no substantive differences in the overall pattern of results compared with the primary RSPL analyses. In contrast, the Bayesian multilevel logistic regression model with non-informative priors yielded a posterior mean variance of 0.71, approximately twice the variance estimated by the primary RSPL model. The 95% highest posterior density (HPD) interval was 0.23–1.41, corresponding to a MOR of 1.58–3.10 Although the RSPL estimate fell within the HPD interval, it was located in the lower part of that interval. In addition, the HPD intervals overlapped across the subsequent models, indicating uncertainty regarding differences in variance estimates between models. However, the overall pattern of change in the estimated between-HSA variance was largely similar to that observed in the frequentist analyses. The main exceptions were the model including GU ownership and the fully adjusted model, for which the estimated between-HSA variance increased rather than decreased. This finding suggests greater uncertainty regarding the contribution of ownership to the observed geographical variation than indicated by the primary analyses. The posterior mean estimates, corresponding 95% HPD intervals, and derived MOR estimates for all Bayesian models (M0-M6) are presented in Additional file 1 (Table A3).

## Discussion

This study examined whether the geographic area of residence was associated with the likelihood that older adults, transported by ambulance, were directly admitted to a GU rather than an ED, and identified factors associated with the observed variability. The descriptive statistics highlight a substantial geographic variation in the prehospital conveyance of patients to GUs across HSAs, where crude HSA-level conveyance rates ranged from 4% to 21% over the eight areas. In the primary analyses, supply-side characteristics, particularly geriatric bed capacity and geographic accessibility, were associated with reductions in the estimated between-HSA variance. Although GU ownership (private vs. public) was not individually significant, it nonetheless contributed to a decrease in variance. Even in the fully adjusted model, residual variation persisted (τ² = 0.2076; ICC = 0.059), reflected by a MOR of 1.54. This implies that although the ICC appears low, which is typical in logistic models because the individual-level variance is fixed at π²/3, otherwise similar patients could experience 1.54 times greater odds of direct GU admission simply by residing in a different HSA. However, the wide HPD interval from the Bayesian models suggests substantial uncertainty regarding the magnitude of the between-HSA variance and the corresponding MOR. This uncertainty was further reflected by the differing pattern observed for the model including GU ownership, which contrasted with the findings from the primary analyses.

Demand-side factors did not explain the variation between HSAs. Although such factors are known to influence healthcare utilisation among older adults [39], adjustment slightly increased between-area variance (τ^2^: 0.35 to 0.38), indicating that patient mix alone does not account for the observed heterogeneity. Nevertheless, the ICC estimates indicated that most of the overall variation in direct admission occurred within rather than between HSAs, suggesting that individual-level factors remain the dominant source of variation despite the presence of geographic differences.

Supply-side elements, such as transportation during office hours, a nearer distance to the GU, and a higher geriatric bed capacity, were identified as correlates of direct GU admission, and the latter two also contributed to estimated reductions in HSA-level variance. Transportation within or out-of-office hours, and proximity to healthcare facility has been raised previously as factors for conveyance to hospitals [40]. Lower conveyance rates outside office hours may reflect operational constraints, such as reduced diagnostic support or resource availability outside regular hours. Additional non-medical factors influencing prehospital decision-making, such as limited organizational support or EMS personnel’s perceived responsibility for patient safety, have been noted in previous research [4]. An incorrect decision to not convey an older person could lead to adverse or health-threatening events, at the same time as a conveyance decision to an ED may lead to unnecessary use of healthcare resources [4]. Geographic accessibility to a GU decreased the estimated between HSA-variance by 26% (ICC = 0.079), consistent with literature showing that ambulances frequently convey patients to the nearest facility when distance differentials widen [41]. Nonetheless, it is also reported that up to one-third of ambulances may bypass the nearest ED, suggesting that multiple logistical and organizational factors may contribute to destination choices [41]. In our data, higher numbers of staffed geriatric beds were associated with direct admission to GUs, reducing the estimated between-HSA variance by one-third. However, prior work on hospital bed supply underscores the risk of supply-sensitive utilisation, emphasising the need for careful capacity regulation [42]. It is also important to note that causal inference cannot be established from the present study. Given the small number of HSAs and the resulting imprecision of area-level estimates, the observed association should be interpreted cautiously and regarded as exploratory. Even if bed supply correlates with utilization, policy responses must avoid simple capacity expansions that risk supply-induced demand [15], [18], [42–44].

Aligning these findings with planning of geriatric care may impact location of geriatric units as well as a thorough review of differences that may impact non-conveyance during out-of-office hours, such as e.g. capacity in terms of both staff and access to diagnostics and lab tests. This ought to be studied more thoroughly. Regarding generalizability, it should be noted that the study was conducted in a healthcare system with public funding of both private and public healthcare providers as well as ambulance care. The experiences are probably more relevant in densely populated regions with a range of acute hospitals and independent GUs. The choice to convey older acute ill patients between EDs at acute hospitals and GUs is also a relatively new approach to the care of this patient group. Despite this, the study can provide guidance on how demand- and supply side may contribute to variations in access to this form of care.

## Limitations

Even if this study used the entire population-based sample from the largest region of Sweden, the number of clusters were few, which warrants caution as estimates may be biased [33]. The estimated between-area variance and corresponding MOR should therefore be interpreted with caution. While the MOR decreased across successive adjustment models, the Bayesian robustness analysis indicated substantial uncertainty around the variance component, suggesting that differences in MOR between models may be less distinct than implied by the point estimate alone. Furthermore, the posterior mean variance estimated in the Bayesian baseline model was higher than the corresponding variance estimates obtained in the primary RSPL analyses, a pattern consistent with previous research indicating that variance estimates in multilevel logistic regression models with few clusters may be sensitive to the choice of estimation method [33]. Consequently, both the magnitude of the residual geographical variation and the observed reduction across models should be interpreted as suggestive rather than conclusive. Furthermore, the study lacks measures capturing organizational and protocol-level factors (e.g., availability of equipment and laboratory tests, real-time diversion, bed management practices, and capacity constraints), which may influence ambulance routing and acceptance rates at GUs. Information on cognitive impairment and functional status was also unavailable in the register data. Although dementia is partially captured within the CCI, neither cognitive status nor functional ability could be comprehensively assessed. These factors may influence both eligibility for direct GU admission and conveyance decisions and may therefore represent residual sources of unexplained variation. The absence of these measures may limit the interpretation of factors associated with access to direct geriatric care. Moreover, as exact clinical eligibility for direct admission could not be determined from the available register data, some patients who would not have been considered suitable candidates for direct admission may have been included in the study population. This would most likely increase the number of potentially eligible patients and consequently lead to an underestimation of the crude direct admission rate. However, the magnitude of this potential bias could not be quantified from the available data.

Another potential limitation is that distance metrics were calculated from patients’ residential addresses rather than their actual pickup locations. The spatial circumstances of the pickup event may therefore differ from those implied by the residential address, potentially resulting in exposure misclassification. Because information on pickup location was unavailable, the extent of this misclassification could not be quantified. Finally, our models focused on conveyance rather than patient outcomes, and we did not assess whether direct GU admission improved functional status, readmissions, or mortality in this cohort. However, it has been argued as advantageous in other settings [5], [6].

## Conclusions

This study demonstrated considerable geographic variation in direct ambulance conveyance to GUs among older adults. The finding that similar older adults face different probabilities of reaching geriatric-specialty care depending on HSA raises equity as well as efficiency concerns within the emergency care pathway. The results showed that direct admission to acute geriatric care units was associated with ambulance conveyance during office hours and a nearer distance to the GU, even after controlling for other demand- and supply-side factors. Between-HSA differences were partly associated with geographic accessibility, geriatric bed capacity, and potentially GU ownership, although the latter finding was not consistent across estimation approaches. While both individual- and area-level factors contributed to the observed variation, residual disparities remained after adjustment, suggesting the influence of unmeasured contextual or organizational factors. However, most of the overall variation in direct admission occurred within HSAs rather than between them, highlighting the importance of individual-level factors alongside area-level influences. Given the small number of HSAs, associations involving area-level characteristics should be interpreted cautiously and regarded as exploratory. Taken together, these findings suggest that the organization and capacity of geriatric services may play a role in supporting more consistent and equitable care pathways. Such efforts may improve healthcare delivery for older adults while promoting efficient use of resources and sustained access to appropriate care.

## Supplementary information

Below is the link to the electronic supplementary material.

Supplementary Material 1

## Data Availability

The data that support the findings of this study are available from Healthcare Committee Department at Region Stockholm, but restrictions apply to the availability of these data, which were used under license for the current study, and so are not publicly available. Data are however available from the authors upon reasonable request and with permission from the Ethical Review Board and the Healthcare Committee Department at Region Stockholm.

## Abbreviations

AGQ: Adaptive Gaussian quadrature
CCI: Charlson comorbidity index
CI: Confidence interval
ED: Emergency department
ESS: Emergency symptoms and signs
GU: Geriatric unit
HPD: Highest posterior density
HSA: Hospital service area
ICC: Intraclass correlation coefficient
M: Model
MOR: Median odds ratio
NACA: National Advisory Committee for Aeronautics
OR: Odds ratio
PCV: Proportional change of variance
RETTS: Rapid Emergency Triage and Treatment System
RSPL: Residual subject-specific pseudo-likelihood
SE: Standard error
SES: Socioeconomic status
