# Supplementary material for: Disparities in direct access to acute geriatric care – an observational analysis of geographic variations of ambulance conveyance
Source: BMC Geriatr. 2026 Oct 2;26:1244. doi: 10.1186/s12877-026-08370-1 (PMC13632365; doi:10.1186/s12877-026-08370-1)
Supplement: Supplementary file 1 [file 12877_2026_8370_MOESM1_ESM.pdf]

Additional file 1

Tables

Table A1. Multilevel logistic regression models of demand side factors, RSPL and AGQ estimations

	M1.1 RSPL	M1.2 AGQ
Intercept (Estimate)	-2.43	-2.44
**MEASURES OF ASSOCIATION, OR (CI)**		
**INDIVIDUAL CHARACTERISTICS**		
**Age groups**		
75-84 years (vs 65-74 years)	2.58 (1.90-3.51)*	2.58 (1.90-3.52)*
85-94 years (vs 65-74 years)	4.49 (3.32-6.06)*	4.49 (3.32-6.07)*
≥95 years (vs 65-74 years)	8.33 (5.67-12.24)*	8.35 (5.68-12.26)*
**Sex**		
Female (vs male)	1.16 (0.97-1.39)	1.16 (0.97-1.39)
**Lifestyle/SES**		
Medium advantaged area (vs Highly advantaged area)	1.39 (1.09-1.76)*	1.39 (1.069-1.76)*
Disadvantaged area (vs Highly advantaged area)	1.26 (0.99-1.61)	1.27 (0.99-1.61)
**Living situation**		
Institution (vs Home)	0.31 (0.20-0.48)*	0.31 (0.20-0.48)*
**Days hospitalized previous year**		
Less than two weeks (vs None)	1.47 (1.16-1.87)*	1.47 (1.16-1.87)*
Two weeks or more (vs None)	2.42 (1.96-3.00)*	2.42 (1.96-3.00)*
**Comorbidity (CCI)**		
Mild comorbidity (vs No comorbidity)	1.33 (0.93-1.91)	1.34 (0.93-1.91)
Moderate comorbidity (vs No comorbidity)	1.31 (0.91-1.89)	1.31 (0.91-1.89)
Severe comorbidity (vs No comorbidity)	1.16 (0.80-1.68)	1.16 (0.80-1.68)
**MEASURES OF VARIATION OR CLUSTERING**		
HSA variance, τ^2 (SE)	0.38 (0.220)	0.329 (0.179)
ICC	0.104	0.091
MOR	1.80	1.73

RSPL= Residual subject-specific pseudo-likelihood; AGQ= Adaptive Gaussian quadrature

Table A2. Multilevel logistic regression models using adaptive Gaussian quadrature estimation

	M0	M1	M2	M3	M4	M5	M6
**MEASURES OF ASSOCIATION, OR (CI)**							
Intercept (Estimate)	-2.44						
Individual characteristics		*NR*	*NR*	*NR*	*NR*	*NR*	*NR*
**INDIVIDUAL LEVEL**							
**Admission time**							
Office hours (vs out-of-office hours)			1.77* (1.49-2.11)				1.79* (1.50-2.14)
**Geographic accessibility**							
GU closer (vs equal distance)				1.45* (0.30-0.80)			1.41* (1.06-1.87)
ED closer (vs equal distance)				0.49* (0.30-0.80)			0.48* (0.30-0.78)
**HSA LEVEL**							
**GU ownership**							
Private (vs public)					2.26* (1.07-4.80)		1.38 (0.70-2.72)
**Geriatric bed capacity**							
Beds per 1000 older population (continuous)						2.01* (1.18-3.41)	1.60 (0.96-2.67)
**MEASURES OF VARIATION OR CLUSTERING**							
HSA variance, τ^2 (SE)	0.302 (0.165)	0.329 (0.179)	0.329 (0.179)	0.240 (0.134)	0.200 (0.114)	0.171 (0.096)	0.121 (0.070)
PCV(M0)[a]	Ref	-8.9%	-8.9%	20.5%	33.8%	43.4%	59.9%
PCV(M1)[b]		Ref	0	27.1%	39.2%	48.0%	63.2%
ICC	0.084	0.091	0.091	0.068	0.057	0.049	0.035
MOR	1.69	1.73	1.73	1.60	1.53	1.48	1.39

OR= Odds ratio; CI= Confidence interval; NR=Not reported (data available in Additional file 1, Table A1); τ^2 = tau-squared; SE= Standard error; PCV= Proportional change of variance; ICC=Intraclass correlation; MOR = Median odds ratio

[a]PCV relative to the baseline model (M0)

[b]PCV relative to the demand-adjusted model (M1)

*$p<0.05$

Table A3. Robustness analysis of area-level variation using Bayesian models with non-informative priors, presented as posterior mean estimates and 95% HPD intervals

MEASURES OF VARIATION OR CLUSTERING							
	**M0**	**M1**	**M2**	**M3**	**M4**	**M5**	**M6**
Posterior mean τ^2	0.71	0.74	0.73	0.66	0.76	0.69	0.75
HPD interval	0.23-1.41	0.23-1.48	0.24-1.42	0.22-1.29	0.23-1.63	0.20-1.37	0.19-1.66
Approximate MOR	2.23	2.27	2.26	2.17	2.30	2.21	2.28
Approximate HDP interval of MOR	1.58-3.10	1.58-3.19	1.59-3.12	1.56-2.95	1.57-3.38	1.53-3.06	1.52-3.42

τ^2 = tau-squared; HPD = Highest posterior density; MOR = Median odds ratio

Figures

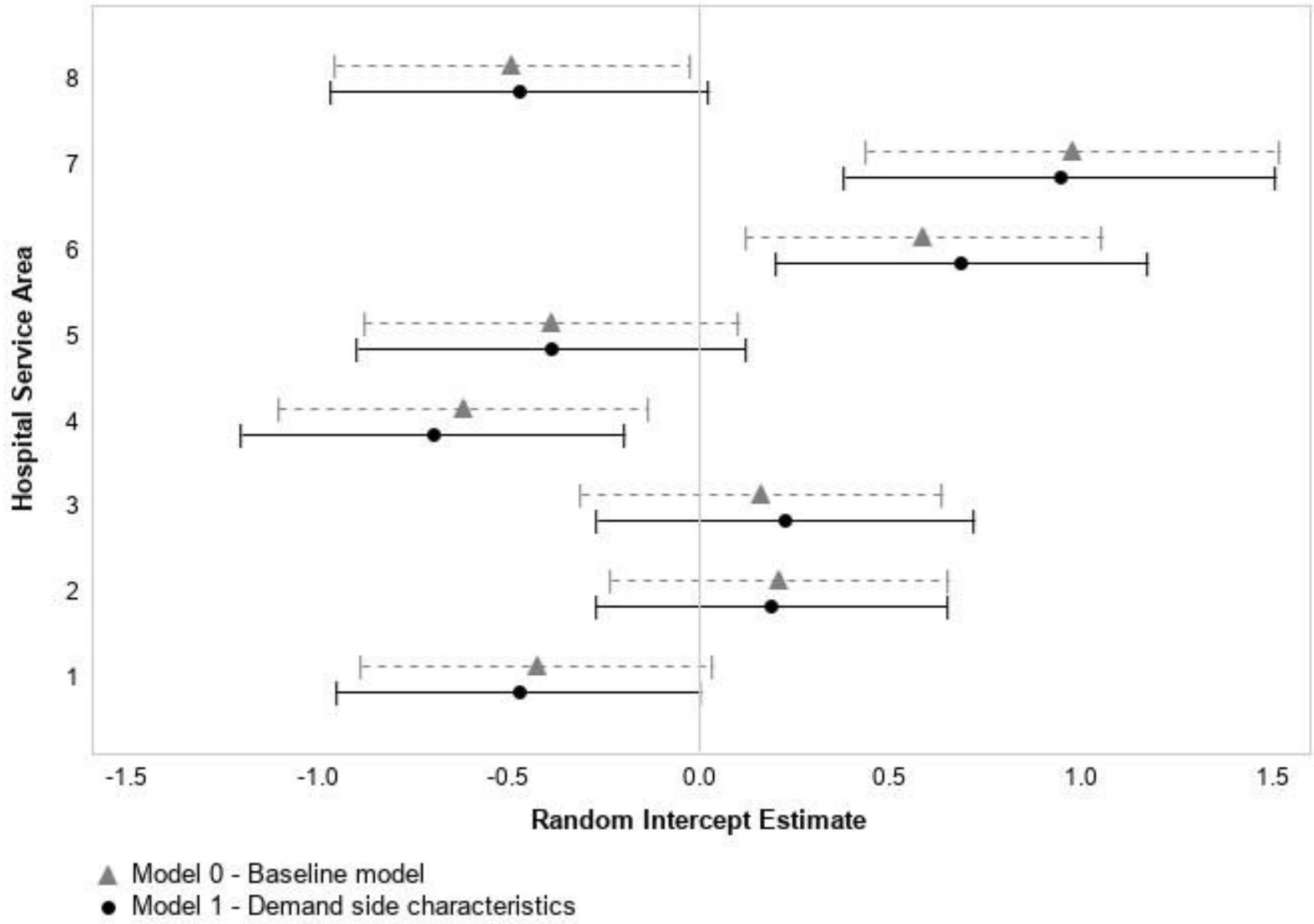

Figure A1. Comparison of Model 0 and Model 1. Random intercept estimates and confidence intervals (CI 95%) for each HSA respectively

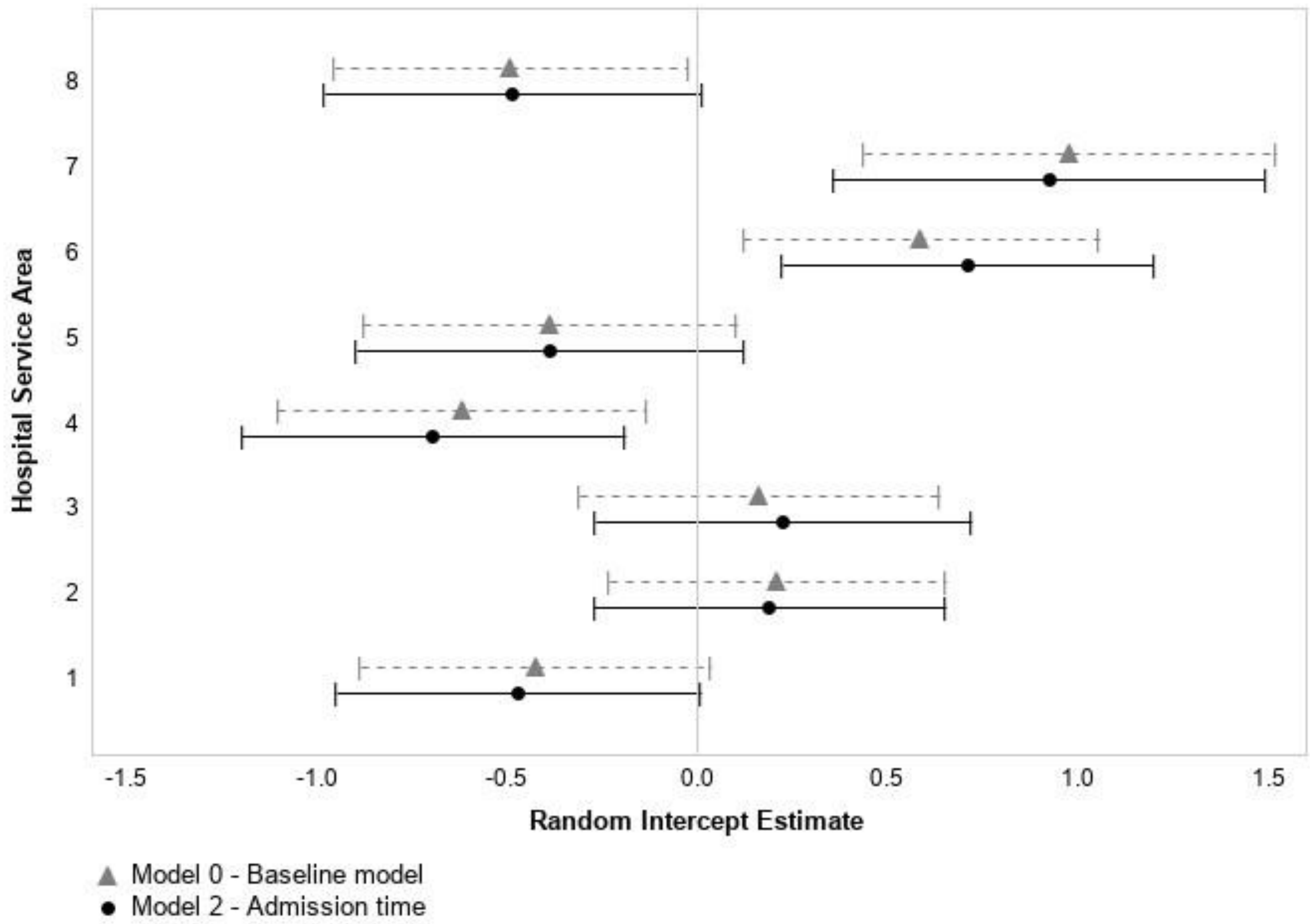

Figure A2. Comparison of Model 0 and Model 2. Random intercept estimates and confidence intervals (CI 95%) for each HSA respectively

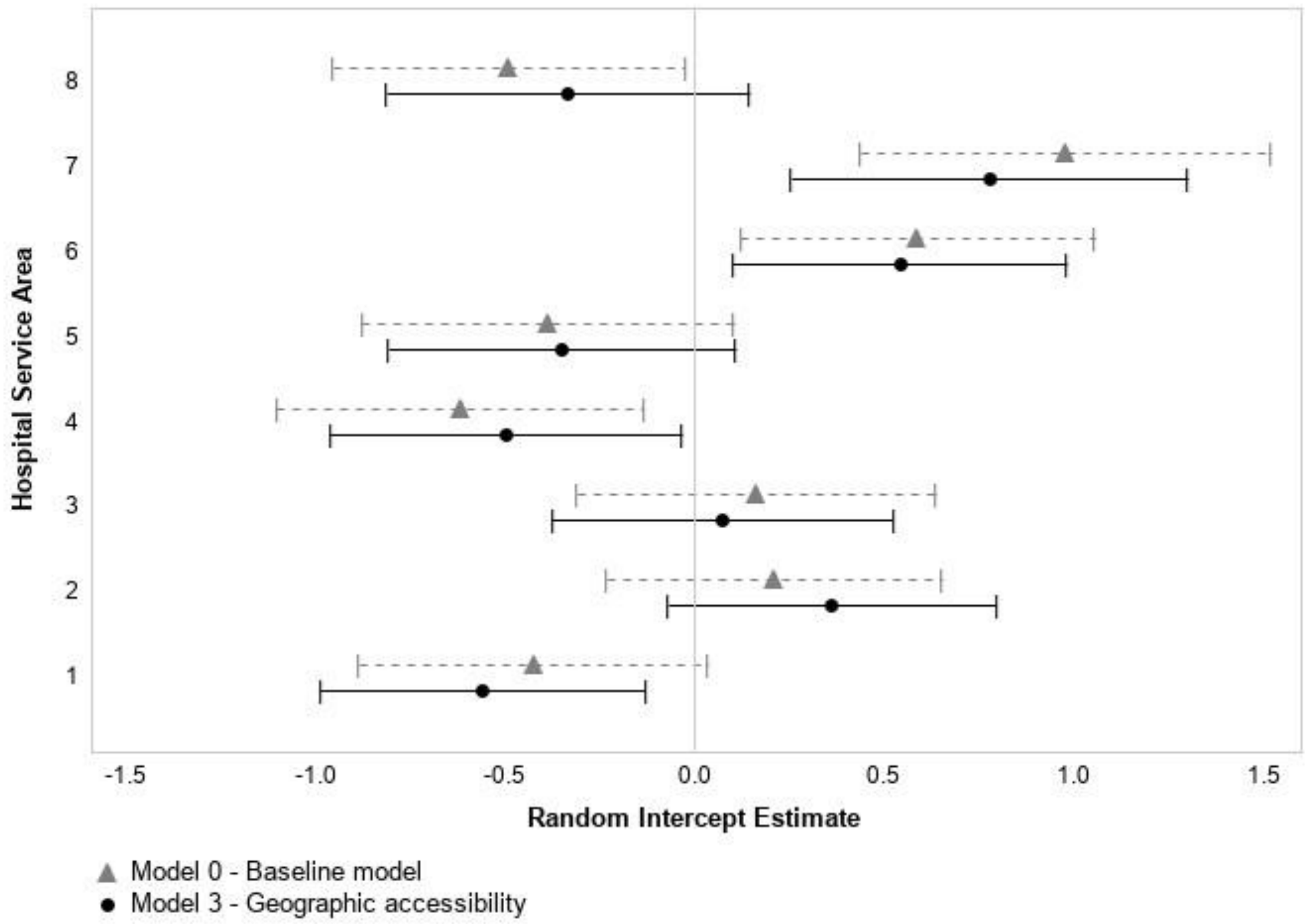

Figure A3. Comparison of Model 0 and Model 3. Random intercept estimates and confidence intervals (CI 95%) for each HSA respectively

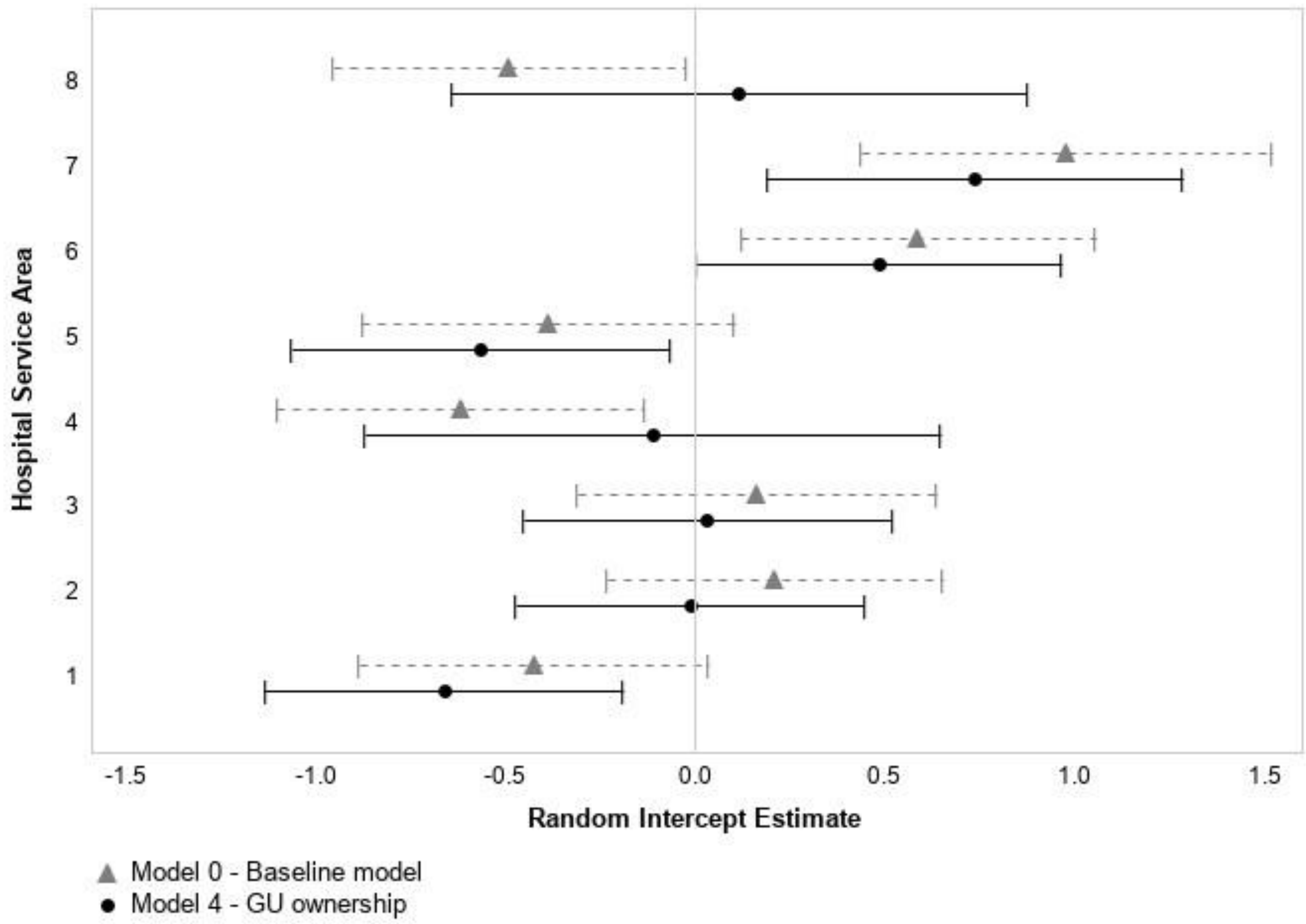

Figure A4. Comparison of Model 0 and Model 4. Random intercept estimates and confidence intervals (CI 95%) for each HSA respectively

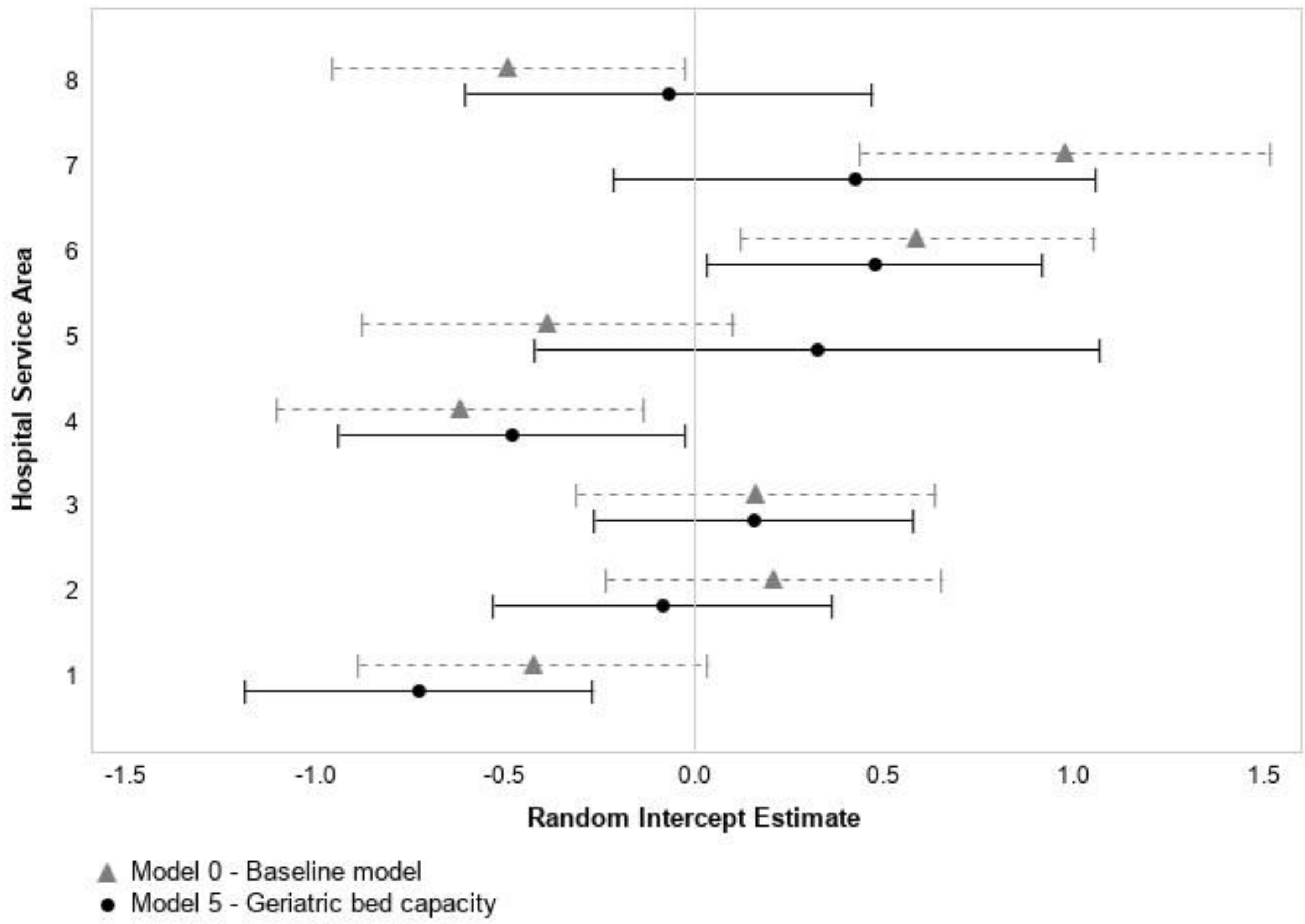

Figure A5. Comparison of Model 0 and Model 5. Random intercept estimates and confidence intervals (CI 95%) for each HSA respectively
